# Retraction Notice to: miR-93 Promotes the Growth and Invasion of Prostate Cancer by Upregulating Its Target Genes TGFBR2, ITGB8, and LATS2

**DOI:** 10.1016/j.omton.2026.201362

**Published:** 2026-10-17

**Authors:** Jia-Ji Liu, Xuan Zhang, Xiao-Hou Wu

## Main text

(Molecular Therapy: Oncolytics *11*, 14–19; December 2018)

*Molecular Therapy Oncology* is retracting this article at the request of the editor-in-chief. An editor from another journal communicated concerns to the editorial office. Their investigation drew attention to similarities between the figures in this article and in a retracted article by unrelated authors in *FEBS Letters* (Xu et al., 2013, 3166–74, https://doi.org/10.1016/j.febslet.2013.05.069). Image analysis performed by the editorial office revealed evidence of image duplication involving Figures 3C, 4A, and 6B of this *Molecular Therapy Oncology* article. This reuse of data without appropriate attribution represents a severe abuse of the scientific publishing system. The authors did not respond to the original notice of retraction.

